# Influence of PBF-LB Process Atmosphere on the Fatigue Strength of Hot Isostatically Post-Densified Duplex Steel Parts Produced via the Shell Core Approach

**DOI:** 10.3390/ma16114014

**Published:** 2023-05-27

**Authors:** Anke Kaletsch, Markus Sondermann, Markus Mirz, Felix Radtke, Christoph Broeckmann

**Affiliations:** 1 Institute for Materials Applications in Mechanical Engineering (IWM), RWTH Aachen University, 52062 Aachen, Germany; 2 Institute of Applied Powder Metallurgy and Ceramics at RWTH Aachen e.V. (IAPK), 52062 Aachen, Germany

**Keywords:** additive manufacturing, laser powder bed fusion, PBF-LB, hot isostatic pressing, post-densification, process gases, combination AM and HIP, shell–core, productivity enhancement

## Abstract

Laser-based additive manufacturing is a great manufacturing technology for producing parts of any geometry. To also increase the strength and reliability of parts produced via powder bed fusion with laser beam (PBF-LB), hot isostatic pressing (HIP) is often used to densify residual porosity or lack-of-fusion defects. When components are post-densified via HIP, they do not require a high density beforehand, only a closed porosity or a dense shell. By building up samples with increased porosity, the PBF-LB process can be accelerated and productivity increased. HIP post-treatment gives the material its full density and good mechanical properties. However, with this approach, the influence of the process gases becomes important. Either argon or nitrogen is used in the PBF-LB process. It is assumed that these process gases are trapped in the pores and thus have an influence on the HIP process and also the mechanical properties after HIP. In this study, the influence of argon and nitrogen as process gases on the properties of duplex AISI 318LN steel after powder bed fusion with laser beam and hot isostatic pressing is investigated for the case of very high initial porosities.

## 1. Introduction

Additive manufacturing (AM) processes are of great interest and the subject of comprehensive research. One of the most commonly used AM technologies is powder bed fusion with laser beam (PBF-LB). Components built by PBF-LB can already be found in various technical applications such as aerospace, motorsports, or prototyping. Nevertheless, in many areas, PBF-LB-manufactured parts are not yet used in existing applications because the suitability for serial production is often limited [1]. This is based on different reasons: on the one hand, the processing time for high quantities or big parts is not yet economical, and on the other hand, the reliability of the manufactured components is often insufficient due to internal defects and anisotropic microstructure [2,3,4]. A popular way to optimize the mechanical properties of additively manufactured components is hot isostatic post-densification. Hot isostatic pressing (HIP) is an established process in powder metallurgy, where materials of the highest quality can be produced [5]. The obtained microstructure is homogeneous and pore-free. Thus, using HIP post-processing, additively manufactured components can be enormously optimized, in particular concerning their fatigue strength [6,7,8,9,10]. At the same time, by using HIP, a productivity increase within the PBF-LB process is possible because components subjected to HIP post-treatment do not require a high quality before HIP. The production of high-quality samples with low porosity in PBF-LB requires long process times. If samples are intended for HIP post-densification, higher porosity is allowed, which gives the chance to save process time. Equation (1) shows that the theoretical build rate in the PBF-LB process can be approximated by the product of scan velocity *v_s_*, hatch distance *d_h_*, and layer thickness *t_l_*.

(1) $$Build\;rate=v_{s}\times t_{l}\times d_{h}$$

By assuming a constant laser power *P_L_*, an increase in each parameter will lead to a faster process, but simultaneously reduces the energy input and thereby increases the porosity (see Equation (2)).

(2) $$E_{d}=\frac{P_{L}}{v_{s}\times t_{l}\times d_{h}}$$

If the energy density *E_d_* is too low, individual regions are not sufficiently molten, and binding errors such as “lack-of-fusion” defects occur [11].

**Figure 1:**
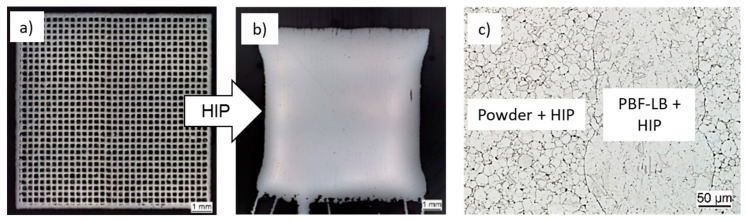
Representation of the shell core concept: (**a**) sample with high porosity in the core with dense shell (after PBF-LB, cavities have loose powder inside); (**b**) fully densified sample after HIP; (**c**) different areas in the microstructure after HIP.

Studies that are already using approaches to increase the productivity of PBF-LB are known from the literature [12,13,14,15,16]. Leicht et al. [14] enhanced the productivity by increasing the layer thickness from 20 µm to 80 µm, resulting in a relative density higher than 99.9% and good tensile strengths, but a slight decrease in yield strength. In another study, the same group showed that by adjusting the hatch distance and scanning speed, the build rate could be increased by 20% and the material still maintained good properties [15]. A shell core approach has already been used in a study by De Formanoir et al. [16]. Here, an increased porosity was introduced into the core of the sample by enlarging the layer thickness. The shell of the sample, printed with a smaller layer thickness, was virtually dense. However, in these studies, the samples were not post-densified by HIP. Approaches with HIP post-densification to achieve high strength and reliable properties were described by other groups. In our own studies, an increase in scanning speed [17] and an increase in hatch distance [18] have been used to accelerate the PBF-LB process. An increased scanning speed has also been used by Herzog et al. [19], where the resulting porosity of about 5% was then densified by HIP. A porosity of up to 5% can be densified in HIP without an additional building of a dense shell since it can be assumed as closed porosity. If higher porosity needs to be densified by HIP, a dense shell must be built around the porous core in the PBF-LB process, as shown in Figure 1a. Core porosities of up to 10% which were covered by a dense shell were successfully densified in [18]. Depending on the porosity, the specimens show shrinkage after HIP densification (Figure 1b). In order to be able to utilize the geometric advantages of the PBF-LB process and to produce near-net-shape specimens, numerical simulation models were developed which can accurately predict shrinkage during HIP and thus enable initial geometry adjustment [20]. It can also be seen that the resulting microstructure of the individual areas differs, as shown in Figure 1c for the example of the nickel-based alloy Inconel 718.

A shell core concept was also described by Du Plessis et al. [21] where just the dense shell was printed by PBF-LB. The loose powder remaining in the inner cavity was then densified by HIP. In a further study, Du Plessis et al. [22] showed that after HIP, the fatigue strength of the samples produced via this shell core concept was below the fatigue strength of the samples built to full density in the PBF-LB process. The reason might be entrapped argon process gas since pores were still visible after HIP. The higher the initial porosity in the material, the higher the amount of trapped process gas that may counteract compression in the HIP process. The process gases commonly used in the PBF-LB process are argon and nitrogen. Argon has no solubility in metallic crystal structures, so it remains as highly compressed gas pores in the material. These highly compressed argon pores can grow again after the HIP process when the material is exposed to elevated temperatures, such as in heat treatment afterward [23,24,25].

Another possibility would be to use nitrogen as a process gas in the LPBF process, which is often performed especially for austenitic steels because nitrogen shows solubility in the metallic crystal lattice and can even be used for solid solution strengthening in high-nitrogen steels [26,27]. In unalloyed or low-alloyed steels, on the other hand, nitrogen can lead to embrittlement of the material. A group of materials that contains nitrogen as standard are duplex steels.

Duplex steels are highly investigated in the field of laser-based additive manufacturing. They show good processability, and high densities can be achieved in the process [28,29]. Nevertheless, there are some specific aspects to consider: PBF-LB-fabricated duplex steels always require subsequent heat treatment because the rapid solidification in the process prevents austenite precipitation, and a predominantly ferritic microstructure forms instead of the ferritic–austenitic microstructure [30,31].

It should also be noted that at the very high temperatures in the PBF-LB process, evaporation of nitrogen from the melt pool can occur. Since nitrogen stabilizes the austenitic phase, the loss of nitrogen can cause a shift in the temperature of the α/γ-phase equilibrium [32,33].

The present study investigates the shell core concept on the duplex steel AISI 318LN. Here, a special focus is on the influence of the process gases in the PBF-LB process on the microstructure and the mechanical properties after subsequent HIP and heat treatment. Process gas will be introduced into the specimens via increased porosity in the PBF-LB process in order to investigate and understand the influence of different gases on the HIP post-densification and the properties of the material.

## 2. Materials and Methods

### 2.1. Material

In this study, duplex steel AISI 318LN (DIN X2CrNiMoN22-5-3, 1.4462) was used. The powder was gas atomized in a nitrogen atmosphere and provided by “Deutsche Edelstahlwerke GmbH (Krefeld, Germany)”. The chemical composition of the powder was determined by emission spectroscopy, while the carbon and nitrogen contents were measured with LECO CS300 and Bruker GALILEO G8 analyzer, respectively. Table 1 shows the composition of the powder compared with the values according to DIN EN 10088-3:2022-01. All values are within the specifications of the standard. The particle size distribution was d_10_ = 26 µm, d_50_ = 39 µm, and d_90_ = 53 µm.

### 2.2. PBF-LB Processing

In order to investigate the influence of porosity on the HIP process and the mechanical properties after HIP, samples with different relative densities were produced in PBF-LB. In this work, HIP post-densified samples were investigated that had initial relative densities of 80%, 90%, and 99% in the as-built condition.

The shelled PBF-LB samples were fabricated at the Chair of Digital Additive Production (DAP) at RWTH Aachen University on an EOS M290 industrial system equipped with a 400 W fiber laser. Cylindrical specimens with a diameter of 10 mm and a height of 65 mm were built upright in a vertical orientation. The different relative densities were adjusted by varying the hatch distance. If the hatch distance exceeds the width of the molten pool, unmelted powder remains between the molten areas. Thus, with increasing hatch distance, the relative density decreases. The specific process gas remains between the unmolten powder particles. To allow post-densification by HIP, the outer region of the sample was built with high-quality parameters, leading to an almost fully dense and gas-tight shell with a thickness of 1.5 mm. The required process parameters for the PBF-LB process to achieve the relative densities of 80% and 90% were determined in preliminary tests and can be taken from Table 2.

For comparison, fully dense samples were also built, showing a relative density above 99%. These reference specimens were built on a Realizer SLM 100 machine with the same scanning strategy. The process parameters are also listed in Table 2.

### 2.3. Post-Processing

All specimens considered and tested in this study were hot isostatically post-densified, including those whose initial density was already above 99%. The post-densification of the specimens was carried out by the company OWL GmbH in Aachen, Germany. The process parameters for HIP post-densification were as follows: heating with 10 K/min to 1145 °C, followed by a dwell time of 185 min at a pressure of 103 MPa and pressure-less furnace cooling afterward. In order to eliminate possible sigma phase precipitation after the slow cooling in the HIP and to adjust the desired duplex structure, the samples were annealed after the HIP process in a furnace under a nitrogen atmosphere at 1080 °C with a dwell time of 60 min, followed by water quenching.

The samples will be named in the further text according to their initial relative density and the process gas used in the PBF-LB process. The name begins with the process gas and is followed by the relative density before HIP and heat treatment: Ar99, Ar90, Ar80, N99, N90, and N80.

### 2.4. Tensile and Fatigue Testing

**Figure 2:**
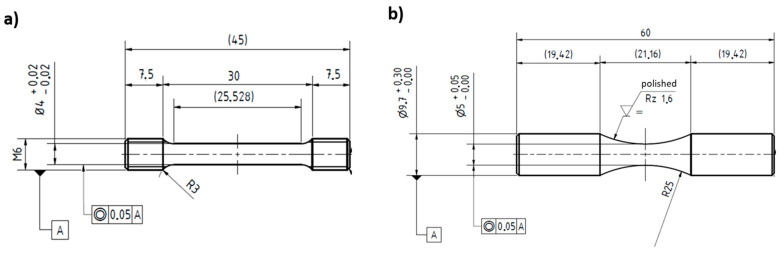
Sample geometry for (**a**) tensile testing and (**b**) fatigue testing.

After HIP, the PBF-LB cylinder specimens were machined to the required test geometries (Figure 2). The final surface treatment was performed after heat treatment.

The tensile tests were performed on a Zwick Roell Zmart Pro materials testing machine (ZwickRoell AG, Ulm, Germany) at a test speed of 0.06 mm/s in accordance with DIN EN ISO 6892:1 [34]. In each condition, between three and six tensile samples were tested.

The rotating bending tests were performed according to DIN 50113:2018-12 [35] at a stress ratio of R = −1 and a frequency of 115 Hz. The staircase method was used to determine the endurance limit. Accordingly, the load level of the individual specimens depends on the result of the previously tested conditions. The ultimate load cycle number was set to 10^7^. For determining the fatigue strength of finite life, the horizontal method was used. The tests were performed on a PUNZ Rapid rotating bending testing machine from Schenck, and the number of specimens tested was at least 21 for each condition. The results were statistically analyzed using SAFD©v5 software (IAPK, Aachen, Germany) [36]. Characteristic fatigue strength data and S-N diagrams were determined for all tested conditions.

### 2.5. Fracture Analyses

After fatigue testing, the fracture surfaces of the specimens were examined using a JEOL-JSM 6500 scanning electron microscope (SEM) to identify the location of crack initiation. For this purpose, overview images of the fracture surfaces were taken, followed by imaging of the crack initiation points at 50×, 200×, 500×, and 1000× magnification. Both secondary electron contrast (SE) and backscattered electron (BSE) images were taken. X-ray microanalysis (energy dispersive X-ray spectroscopy (EDS)) was performed to determine the chemical composition of inclusions.

### 2.6. Microstructure and Density Measurement

After mechanical testing of the specimens, the sample heads were cut in the transverse direction and additionally in the longitudinal direction. Then, the specimens were thermally embedded and subsequently ground and polished.

To investigate the phase fractions and grain size distribution, EBSD measurements were performed on the cross sections of each condition. These were carried out using an EBSD system from EDAX Inc. with a Hikari XP II detector. The analysis was performed with TSL OIM Analysis 7 software.

Image-analytical density measurements were performed on unetched polished cross sections using a Zeiss AxiolmagerM2 optical microscope (LOM) equipped with a SpeedXT core 5 camera from JEN-OPTIK. Three sections were analyzed per micrograph, and the values for porosity and roundness of the pores were determined using ImageJ 1.53 software [37].

### 2.7. Nitrogen, Argon, and Oxygen Measurements

The nitrogen and oxygen measurements were carried out at the Institute of Welding and Joining Technology (ISF) of RWTH Aachen University using hot gas extraction. The measurements were made on four specimens per condition.

The argon content of the samples was measured by gas-phase chromatography. The measurements were performed at Bodycote Specialist Technologies GmbH according to the proposed Swedish national standard for PM-HIP parts [38]. For the argon measurements, between two and four samples per condition were analyzed.

## 3. Results and Discussion

### 3.1. Tensile Testing

**Figure 3:**
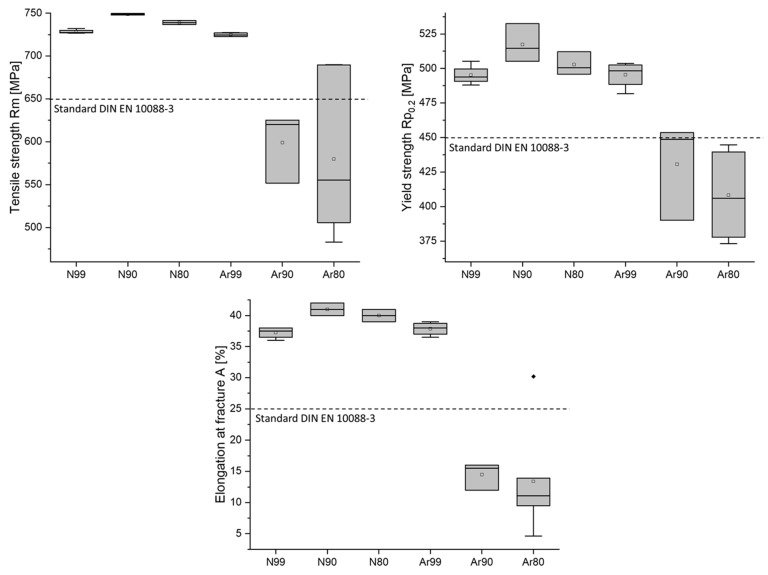
Graphical illustration showing the results of tensile tests on specimens from the different conditions in comparison with the specifications of DIN EN 10088-3 standard. In this box plot, the ends of the boxes and the center line describe the upper, median, and lower quartiles. The statistical average is marked by a small square inside the box. Black rhomboids outside the boxes and the whisker are measured values that were evaluated as outliers.

The mechanical properties obtained from the tensile tests are compared with the specifications of standard DIN EN 10088-3 [39] and with values determined in preliminary tests [40] on fully dense specimens. Figure 3 graphically shows the statistical evaluation of the 0.2% yield strength Rp_0.2_, the tensile strength Rm, and the elongation at fracture A for the individual conditions. The dashed lines indicate the minimum requirements for the mean values according to standard DIN EN 10088-3.

The investigation of the specimens prepared under argon atmosphere shows a clear influence of the initial porosity on both the static strength and the elastic behavior. The tensile strength of the Ar99 specimens reaches an average value of 725 ± 2 MPa and decreases to 599 ± 33 MPa and 580 ± 82 MPa for the Ar90 and Ar80 specimen batches, respectively. The scatter of values is very large for the Ar80 and Ar90 specimens, while all other values scatter less. An influence of initial porosity on tensile strength for all specimens built under argon could be statistically proven via analysis of variance (ANOVA) (*p* = 0.0179). However, by only comparing the specimens with initial porosity Ar80 and Ar90, no significant difference can be found (*p* = 0.7420).

The samples prepared in nitrogen also show a dependence of the initial porosity on the values determined in the tensile test. The average tensile strength is 728 ± 2 MPa for the N99 specimens and increases to 749 ± 1 MPa for N90 and 739 ± 2 MPa for N80. Likewise, yield strength and elongation at break increase. Again, the significance of the difference in tensile strength was proven by ANOVA (*p* = 1.86 × 10^−5^). In contrast to the Ar samples, a difference among the groups is identifiable for the N80 and N90 samples with initial porosity (*p* = 0.0030).

Direct comparison between specimens with the same initial density and different process gases shows approximately the same values for tensile strength, yield strength, and elongation at break for the Ar99 and N99 specimens. No statistical difference in tensile strength could be verified (*p* = 0.0874). However, the results are different for the conditions with lower initial density. There, a significant influence of the process gas is proven both for the specimens with 90% initial density (*p* = 0.0032) and for the specimens with 80% initial density (*p* = 0.0209).

The values specified in the standard for duplex steel AISI 318LN in solution-annealed condition are achieved for all nitrogen specimens regardless of the initial density, while the Ar specimens built by using the shell core approach are below the specifications of the standard for all the characteristic values determined in the tensile tests. This is an indication that entrapped argon gas in the material has an unfavorable influence on the strength. The specimens built to full density under argon, on the other hand, meet the standard.

### 3.2. Fatigue Testing

**Figure 4:**
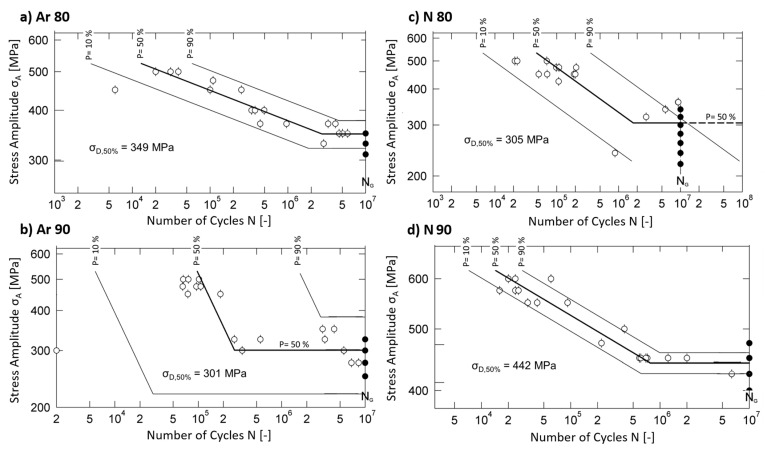
S-N diagrams of the samples, built with different initial densities and in different process atmospheres after HIP and heat treatment: (**a**) built with 80% initial relative density in argon; (**b**) built with 90% initial relative density in argon; (**c**) built with 80% initial relative density in nitrogen; (**d**) built with 90% initial relative density in nitrogen.

Figure 4 shows the S-N curves for the test series built with the shell core approach. The individual lines in the plot of the S-N curves represent the failure probabilities for 10%, 50%, and 90%. The limit for the number of cycles specified for all test series was 10^7^.

**Figure 5:**
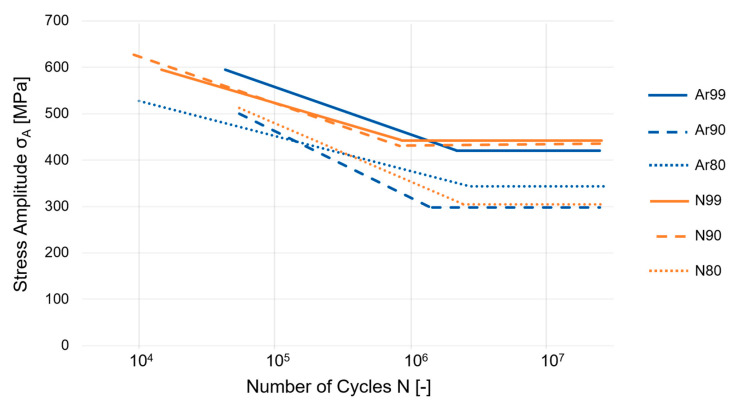
Illustration of all determined S-N curves in comparison.

The fatigue strength of the Ar80 specimens (Figure 4a) was determined to be 349 MPa. The scatter is 28 MPa. In the area of fatigue strength, a constant variation of failures around the 50% S-N curve can be seen. The knee-point of the 50% line is at about 2.7 × 10^6^ cycles. The plot in Figure 4b illustrates the S-N-curve of the Ar90 specimens: Compared to the Ar80 specimens, a much stronger scatter can be seen. In addition, the fatigue strength σ_D_ determined with 301 MPa is lower than the fatigue strength of the Ar80 specimens. The knee-point is at 1.2 × 10^6^ cycles. Figure 4c shows the determined S-N-diagram of the N80 specimens: This test series showed the highest scatter among all the tested conditions and, in addition, a very low fatigue strength. The statistically determined value of the fatigue strength is 305 MPa and the knee-point is 1.7 × 10^6^. Figure 4d presents the S-N-diagram of the N90 specimens: For this test series, a knee-point at 7.8 × 10^5^ and a fatigue strength of 442 MPa with a scatter of 17 MPa were determined. This series showed the lowest scatter of all tested specimens and a fatigue strength close to that of the fully dense reference. Figure 5 summarizes the S-N curves of all investigated conditions for a failure probability of 50%. In addition to the values shown in Figure 4, the results for the Ar99 and N99 conditions are listed.

The results of the fatigue test are summarized in Table 3, where the number of load cycles at the knee-point N_K_ and the fatigue strength σ_D,50%_ are shown.

The investigations reveal a clear influence of the process gas and the initial porosity on the fatigue strength of the specimens. In the case of the specimens produced under nitrogen, the N99 and the N90 specimens show an equivalent fatigue strength, which is at a high level. Here, the fatigue strength of the N90 specimens even exceeds that of the initially fully dense built Ar99 specimens. For the N80 specimens, however, a significant drop can be observed compared to the N90 and the N99 specimens. The calculated fatigue strength for these specimens is only 305 MPa.

For the argon-built specimens, this drop in fatigue strength is already evident for the Ar90 specimens, where the fatigue strength is even lower than for the Ar80 specimens. The scatter of the Ar90 samples, together with that of the N80 samples, is the largest of all conditions.

It is obvious that the determined fatigue strength was higher when the samples were produced under a nitrogen atmosphere than under argon. The reason for this will be clarified in the next subsections with the help of fracture and microstructural analyses.

### 3.3. Fracture Analysis

**Figure 6:**
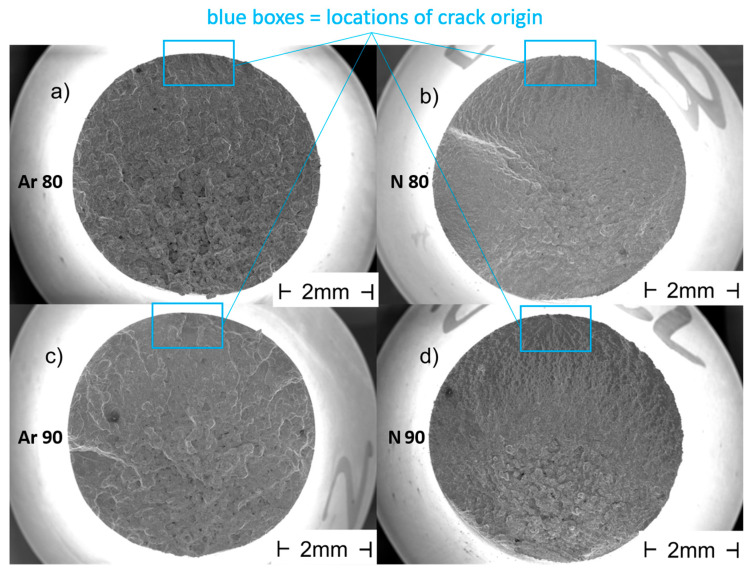
Overview images of the fatigue fracture surfaces of representative samples: (**a**) built with 80% initial relative density in argon; (**b**) built with 80% initial relative density in nitrogen; (**c**) built with 90% initial relative density in argon; (**d**) built with 90% initial relative density in nitrogen.

Figure 6 shows exemplary overview images of the fracture surfaces of samples with an initial density of 80% and 90% manufactured under argon and nitrogen.

**Figure 7:**
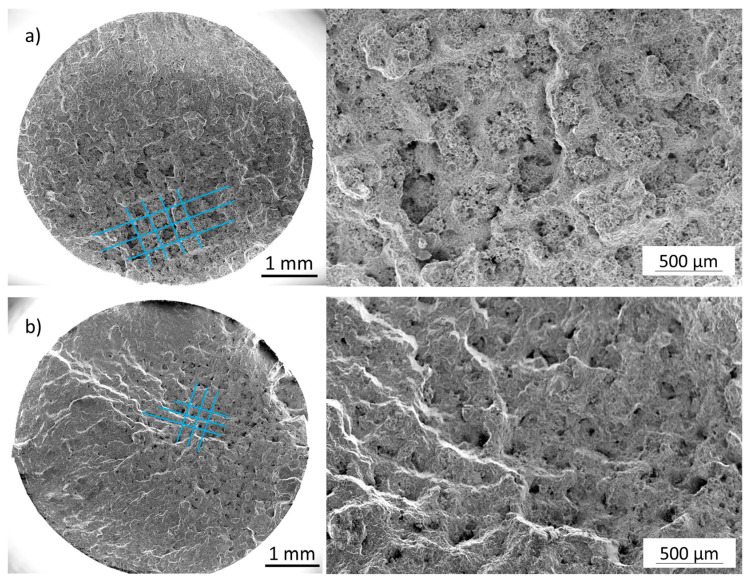
Overview of fracture surfaces that clearly show the PBF-LB scanning strategy (blue lines) in the area of the final forced fracture: (**a**) Ar80 specimen; (**b**) Ar90 specimen.

The SEM images shown in Figure 6 are each oriented so that the crack initiation point is located at the upper point of the specimen. A comparison of the used process gases shows on the one hand that the nitrogen specimens (Figure 6b,d) show typical fatigue fracture surfaces with a well-visible crack propagation, while this is less clear for the Ar specimens (Figure 6a,c). On the other hand, for the specimens produced under argon, the lattice structure generated by the PBF-LB process (compare Figure 1a) is clearly visible on the remaining final fatigue fracture surfaces despite post-compression by HIP and subsequent heat treatment. A detailed illustration of this aspect is provided in Figure 7. In particular, the sample from the Ar80 series reveals the lattice structure melted via PBF-LB. The areas in between, where the HIP consolidated powder is present, have a very porous appearance. The following hypothesis can be used as an explanation: During the HIP process, the argon cannot diffuse into the matrix of the duplex steel due to its insolubility, and it is highly compressed by the high HIP pressure. The powder particles in the cavities will (partially) sinter, and the argon will concentrate in certain areas, possibly at the interface with the areas melted by PBF-LB. In areas that are not entirely sintered, delamination may occur when the compressed argon gas expands during the temperature rise of the pressure-less heat treatment after HIP.

For the sample in Figure 7a, the hatch distance in the PBF-LB process was 600 µm. Due to shrinkage during HIP consolidation, the distance in Figure 7a is slightly smaller than 500 µm. In addition, in Figure 7b, uniformly arranged porous structures can be seen, which must be the former cavities with loose powder.

The nitrogen specimens do not show this characteristic. This may be an indication that the high amount of compressed argon either hinders good bonding of the powders to the area melted in PBF-LB or that during the subsequent pressure-less heat treatment, re-growth of the compressed argon gas leads to delamination of the interfaces.

**Figure 8:**
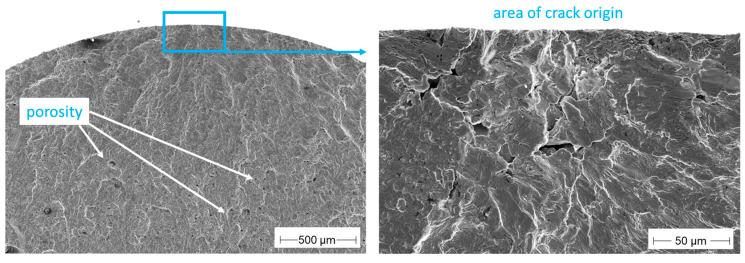
Large irregularly shaped pore in an Ar80 sample, 475 MPa, N = 109,000.

In half of all specimens built under argon, pores are found to be the origin of the fracture. Figure 8 shows the crack-initiating point of an Ar80 specimen. In addition to the large irregularly shaped pore at the specimen edge, where the crack started, many other pores are visible on the fracture surface.

**Figure 9:**
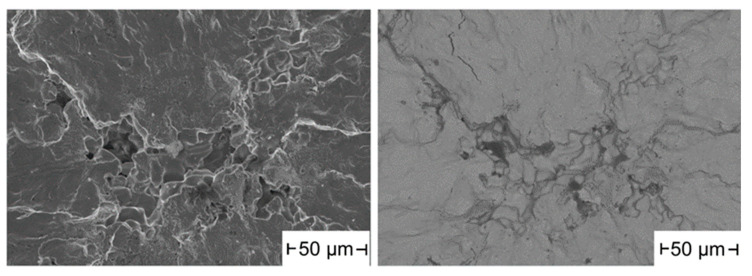
Large irregular formed pore in an Ar90 sample as SE (**left**) and BSE (**right**) image; 475 MPa, N = 65,000.

Figure 9 shows another pore as a crack origin in an Ar90 specimen. In the BSE image (right image) some dark spots are visible, which are oxides inside the pore. In addition, clearly visible are powder particles in the defect which are not completely densified. Such big and irregularly formed pores could be detected almost exclusively in samples prepared under argon. This is an indication that argon porosity grew again during subsequent pressure-less heat treatment.

**Figure 10:**
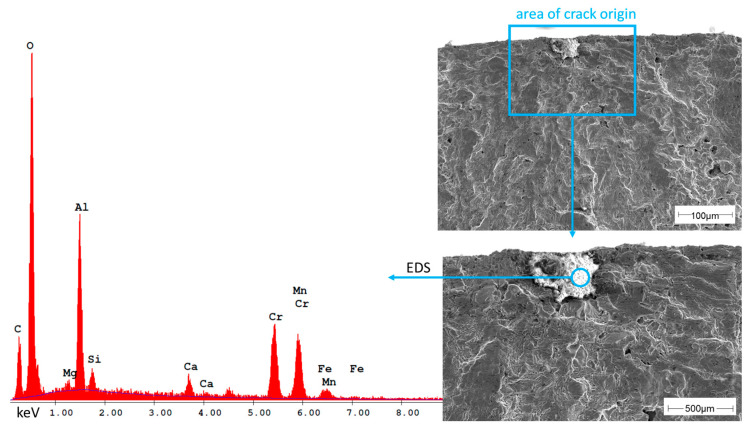
Non-metallic inclusion in an Ar80 sample and EDS spectrum showing the elements detected in the inclusion.

Nevertheless, in addition to large pores, non-metallic inclusions in near-surface areas were also identified as the cause of cracks in 50% of the samples produced under argon. One example is shown in Figure 10.

The EDS spectrum clearly shows that the inclusion is mainly an aluminum-rich oxide. Since the duplex steel used in this study does not contain aluminum, it can be assumed that these impurities in the powder are from the powder atomization process. In addition to aluminum, small amounts of magnesium and calcium are found as well, which are also not alloying elements in duplex steel. All other found elements are alloying elements of the steel used.

**Figure 11:**
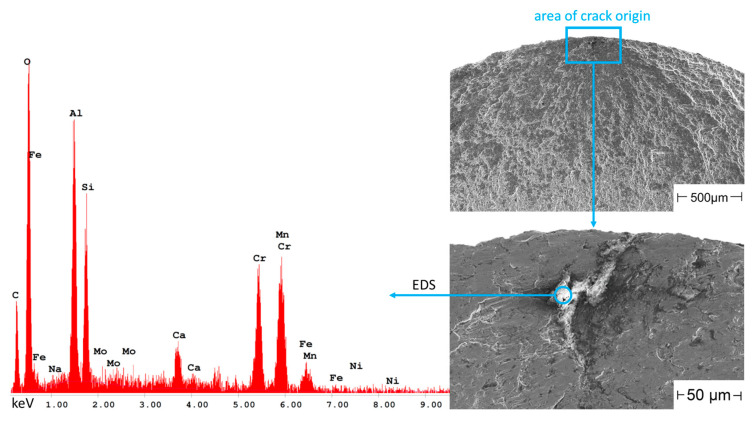
Non-metallic inclusion in an N90 sample and EDS spectrum showing the elements detected in the inclusion.

**Figure 12:**
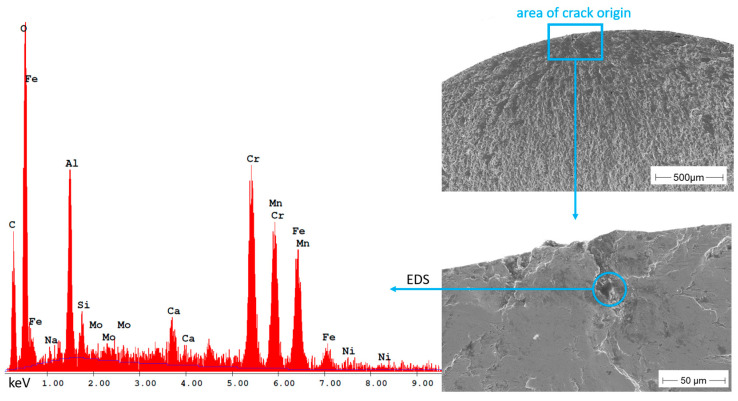
Non-metallic inclusion in an N80 sample and EDS spectrum showing the elements detected in the inclusion.

In comparison to the specimens built under argon, crack initiation in the specimens built under nitrogen occurred almost exclusively at such non-metallic inclusions. Examples thereof are shown in Figure 11 for a sample from the N90 series and in Figure 12 for a sample from the N80 series.

The inclusion in Figure 11 is as well a very aluminum-rich oxide, which at the same time also contains large amounts of silicon. The crack-initiating defect in the N80 sample in Figure 12 also consists of an aluminum-rich oxide inclusion, but this defect could also be a large pore connected with the surface. For the examination in Table 4, due to the high amount of aluminum and oxygen, this defect was considered as an inclusion.

Table 4 gives an overview of the results of the fracture surface analysis. For each series, a certain number of samples was examined (columns 1 and 2). First, the number of specimens with multiple crack paths was determined (columns 3 and 4). Then all crack-inducing defects of the individual sample series were counted and it was analyzed how many of these defects were inclusions (absolute) and how many were pores (absolute). The relative amount in % indicates the percentage of all defects found in a sample series that are either inclusions or pores.

The evaluation in Table 4 shows that the crack-initiating defects for the argon specimens were inclusions and pores, while for the nitrogen specimens, mainly inclusions led to fractures. In addition, the Ar90 and N80 specimens, for which the lowest fatigue strength was determined in the rotating bending test, most frequently exhibited multiple fatigue crack initiation sites. Furthermore, the lattice structure printed in the PBF-LB process to adjust the increased initial porosity is clearly visible on the fracture surfaces of the specimens produced under an argon atmosphere. This structure is not visible on the samples produced under a nitrogen atmosphere. In combination with the observed pores, this suggests that gas pores filled with nitrogen can be successfully post-densified by HIP and heat treatment due to the solubility of nitrogen in the metal lattice. Argon-filled defects and pores, on the other hand, expand again after HIP post-densification during pressure-less heat treatment. This expansion of compressed argon pores in subsequent heat treatment could be prevented if the heat treatment is carried out under pressure. A similar study [18] revealed a 3 times higher fatigue strength for shell core samples produced under argon when the heat treatment is not performed under pressure-less conditions afterward, but integrated directly into the HIP cycle and performed under pressure. 

**Table 4:** Summarized results of the fracture surface analysis.

Number of Examined Specimens		Specimens with Multiple Crack Paths		Inclusions		Pores	
		Absolute	Relative (%)	Absolute	Relative (%)	Absolute	Relative (%)
Ar80	8	3	38	5	50	5	50
Ar90	7	4	57	5	46	6	55
N80	11	7	63	15	94	1	6
N90	6	2	33	8	100	0	0

### 3.4. Microstructure

**Figure 13:**
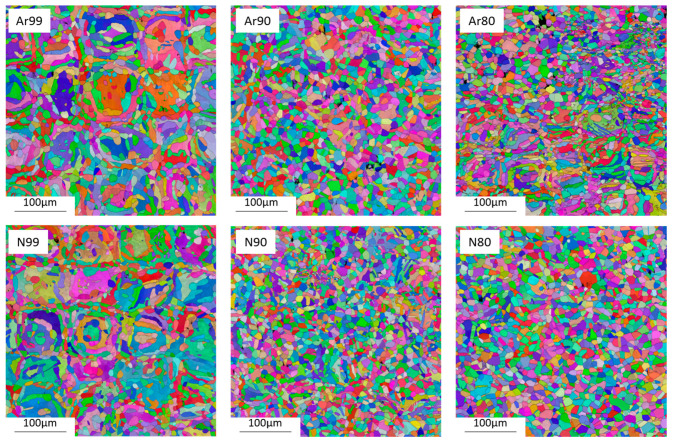
Representation of the microstructure based on an EBSD-IPF measurement as a function of initial relative density and process gas after HIP and heat treatment.

**Figure 14:**
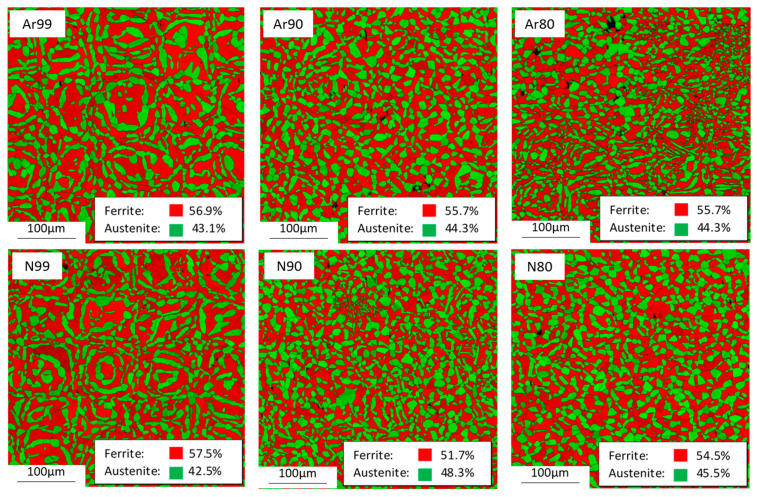
Phase formation based on EBSD measurements as a function of initial relative density and process gas after HIP and heat treatment.

To investigate the influence of process gas and initial density on microstructure and phase formation, EBSD measurements were performed and phase images were obtained. Figure 13 shows inverse pole figure (IPF) images of all investigated conditions, and Figure 14 shows the distribution of the ferritic and austenitic phase fractions based on phase-fit maps.

All specimens exhibit a duplex microstructure with higher proportions of ferrite. The composition of the duplex structure is absolutely identical in the Ar80 and Ar90 samples. Compared to the Ar99 samples, the Ar80 and Ar90 series show a slightly increased austenite content.

**Figure 15:**
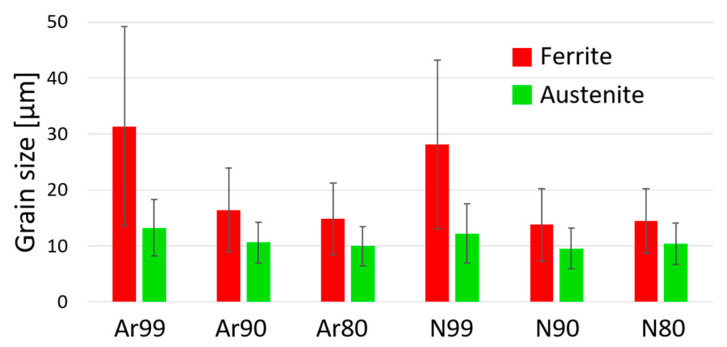
Mean grain size of the different phases after HIP and heat treatment in dependence on the relative initial density and process gas atmosphere.

Like the samples built in argon, the samples produced under nitrogen all exhibit a duplex structure with a higher ferrite content. As the initial density of the samples decreases, the austenite content increases. The largest difference is seen in the N90 specimens, which show the highest overall austenite content of 48%, whereas the N99 specimens have an austenite content of only 42%. Table 5 summarizes the mean grain diameters of the ferritic and austenitic phases depending on the process gas atmosphere and the relative initial densities, and Figure 15 shows the results in a graphical illustration.

A significant difference in mean grain size is observed between fully dense specimens and specimens with initial porosity. This difference can be observed in both the ferritic and austenitic phases but is more pronounced for the ferrite. On the other hand, no difference in the resulting grain size is observed between the samples with initial relative densities of 80% and 90%. In addition, the different process gases used have no major influence on the resulting grain size distribution.

It can be stated that much finer microstructures can be obtained via the shell core method for the material investigated here and that a more balanced austenite/ferrite ratio is achieved. The reason for the finer grain structure in the samples with initial porosity is that they do not have an entirely molten structure, but to a large extent a HIP structure. Moreover, the high initial porosity may interrupt and thus prevent the epitaxial grain growth typical for the PBF-LB process. Similar results have been described by Tosi et al. [41] for specimens porously fabricated via EBM and subsequently post-densified by HIP.

### 3.5. Density Measurement

Three specimens per condition were prepared longitudinally and transversely for metallographic examination. The micrographs were examined and evaluated with ImageJ software in terms of the relative density and roundness of the pores. Table 6 summarizes the results of this evaluation.

It can be seen that all samples after HIP and heat treatment have an average relative density above 99%. The samples with the lower initial densities also have a higher porosity after HIP, but the values differ only slightly. In addition, the samples built porous under nitrogen are slightly denser after HIP and heat treatment than those built under argon. The reason for this may be re-growth of argon porosity during pressure-less heat treatment.

The circularity value compares the shape of the pores with a circle. A circularity of 1 reflects a perfect circle, and a decreasing value is an expression of an irregular shape. The specimens of Ar80, N80, and N90 have a similar circularity of pores between 0.71 and 0.73, while the specimens of Ar90 have a lower value of 0.60. The lower the circularity, the higher the notch effect in the rotating bending test, which in turn has a negative impact on the fatigue strength. This would theoretically be consistent with the results of the fatigue tests, where the Ar90 specimens whose pores had the least roundness also showed the lowest fatigue strength. However, this explanation is not consistent, as the N99 and N90 series have approximately the same fatigue strength, but a significant difference in the circularity of the pores and defects.

**Figure 16:**
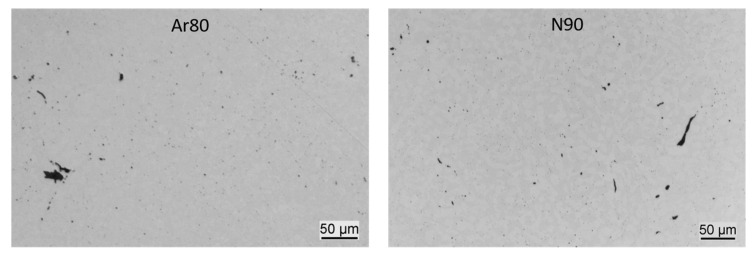
Unetched microsections on representative specimens of the Ar80 (**left**) and N90 (**right**) series.

Compared to the fully dense samples, it can be observed that the roundness of the pores decreases strongly when the shell core approach is applied. The reason for this is that the residual porosity does not consist of round gas-induced pores, but of pores and defects around the former non-fused powder particles and at the interfaces between the fused lattice structure and the regions of loose powder. Therefore, the pores have mostly a very elongated shape, as can be seen in Figure 16. This elongated pore and defect shape can be observed for samples built under nitrogen as well as for those built under argon.

In general, it should be noted that image analysis of pores in micrographs is only two-dimensional, and the largest defect present in the sample, which might lead to failure, is mostly hidden in this type of analysis.

### 3.6. Nitrogen, Argon, and Oxygen Measurements

Table 7 shows the results of the nitrogen, argon, and oxygen measurements. The nitrogen, argon, and oxygen contents of the samples with different initial porosity and process gases were measured and compared with values of fully dense built samples obtained in a previous study. However, in this previous study, no values were provided for the oxygen content of the fully dense built samples. All values are compared to the powder material.

#### 3.6.1. Nitrogen Measurements

The evaluation of the results shows that the nitrogen content of the Ar80 and Ar90 samples is almost identical, but the Ar99 samples have a significantly lower nitrogen content. The reason for this could be a nitrogen loss during the process due to evaporation because the melt has a lower solubility for nitrogen than the solid material. Since the entire material is melted during the production of the Ar99 samples, more nitrogen is lost in this process. For the Ar80 and Ar90 samples, less material is melted, so less nitrogen loss appears.

For the samples built under nitrogen, the N80 samples have a nitrogen content of 1407 ppm, which is comparable to that of the Ar80 and Ar90 samples. Here, a small portion of the nitrogen is lost in the process. Again, the highest nitrogen loss appears for the fully dense N99 samples, where the whole material is melted. However, the loss of nitrogen is less in the fully dense samples produced under nitrogen than in those produced under argon. This may indicate that a nitrogen atmosphere in the PBF-LB process can probably mitigate the nitrogen loss. In addition, the nitrogen content of the N90 samples is even higher (1554 ppm) than the nitrogen content in the initial powder. This could indicate that the material may also absorb nitrogen through the porosity filled with process gas. The nitrogen measurements correlate well with the proportions of the α/γ-phase compositions: the N90 specimens with the highest nitrogen content (1554 ppm) also exhibit the highest percentage of austenite, and the fully dense built specimens with the highest nitrogen loss have lower austenite content than the porous specimens.

#### 3.6.2. Argon Measurements

The argon measurements show that the starting powder has a very low argon content, which is below the resolution limit of 0.049 ppm of the measurement method. This is consistent because the powder should not contain argon, since it was atomized in a nitrogen atmosphere. As argon pick-up in the PBF-LB process can only be expected for the samples built in the argon atmosphere, only these values will be discussed here. It can be seen that all samples pick up argon in the process, even the fully dense samples. The higher the initial porosity, the higher the amount of argon entrapped. In the scanning strategy used here, where the hatch distance is enlarged to increase the porosity, unmolten powder remains in the areas between the fused lattice structures. The powder used in this study has an apparent density of 3.92 g/cm^3^, as given by the manufacturer’s data. With an absolute density of about 7.80 g/cm^3^, the relative apparent density in the powder bed can be assumed to be approximately 51%. It can be expected that the remaining volume is filled with process gas. Thus, the scanning strategy used introduces a large amount of argon into the material, which cannot be removed and is still present in the material even after HIP. This assumption is supported by the measurements in Table 7. At an initial porosity of 10% (Ar90), almost 11 ppm of argon is measured in the material, and at an initial porosity of 20% (Ar80), the argon content is above the maximum value that can be measured with the used method; this means above 20 ppm.

These results fit very well with a study [18] where a porosity of 9.9% was introduced into IN718 samples using the same strategy and the argon content measured in the material after HIP was 10.09 ± 4.85 ppm.

#### 3.6.3. Oxygen Measurements

The oxygen measurements show for both process gases used that the oxygen content increases with a rise in initial porosity. In Figure 8, we have seen that oxides are often found within pores. The reason may be the formation of oxide films on fused surfaces. The samples with increased porosity have a larger area of fused surface inside and may have absorbed more oxygen. This could also be a reason for the decrease in fatigue strength of the N80 specimens compared to the N90 specimens. However, this is only an assumption and has not been proven, especially since the fracture-initiating oxide inclusions were all aluminum-rich and thus presumably originated from powder atomization and not from the process chain investigated here. This point needs further investigation in the future.

## 4. Summary and Conclusions

The shell core concept, in which a dense shell and a porous core are produced in the PBF-LB process and subsequently densified using HIP, is a promising method to increase productivity while achieving good material properties. However, the process gas introduced during PBF-LB into the material via the increased porosity in the core remains in the sample as the gas-tight shell traps it. To determine the effect of different process gases and different initial porosities on the resulting mechanical properties, the initial density of specimens was varied in PBF-LB using different atmospheres. The parameters of the PBF-LB process were adjusted to produce samples with relative densities of 80% and 90% inside the samples. Around the porous core, a gas-tight shell with a thickness of 1.5 mm was built. Argon and nitrogen were used as process gases. Argon cannot be dissolved in the metallic lattice during HIP post-processing, while nitrogen can be dissolved to a certain extent. All samples were subsequently post-densified using HIP and additionally heat-treated to adjust the desired duplex structure. The mechanical properties were determined in static and cyclic tests, and the samples were examined metallographically.

1. The tensile tests show a statistically proven influence of the initial porosity. An influence of the process gas used was verifiable only for the specimens with initial porosity and not for the fully dense specimens. The tensile strength of the specimens produced under nitrogen is above 720 MPa for all conditions, which is in the upper range of the standard for conventionally produced duplex steel AISI 318LN. The individual values vary only slightly, with the highest tensile strength achieved by the specimens built under nitrogen with an initial relative density of 90%. These are also the specimens that show the highest nitrogen content, which was 1554 ppm, even slightly higher than the nitrogen content in the powder, specified by the supplier to be 1500 ppm.
2. The fatigue test shows both an influence of the process gas used and an influence of the initial porosity. For the specimens built under argon with decreased initial densities (80% and 90%), lower fatigue strength values are obtained (301 MPa, 349 MPa). For specimens built under nitrogen, the series with an initial relative density of 90% reach an endurance limit of 442 MPa, which is very close to the value of the fully dense reference (454 MPa). However, for 80% initial relative density, the specimens fall with 305 MPa far behind. We assume that the increased oxygen content found in the N80 samples compared to the N90 samples may result from the formation of oxide skins within the porous structure built in the PBF-LB process. These oxide formations may also influence the fatigue strength. However, this could not be conclusively clarified in this study and is the subject of further investigations.
3. The metallographic examination showed slightly higher porosity after HIP for the samples with higher initial porosity, but the differences are small. In all cases, the relative density after HIP and heat treatment is above 99%. Larger differences were found in pore roundness: all specimens built using the shell core approach have much lower pore roundness than the almost fully dense specimens. The Ar90 samples show the lowest pore roundness with 0.60, while all other samples have roundness values above 0.71. The Ar90 specimens also showed the lowest fatigue strength. However, it cannot be assumed that the notch effect due to irregularly shaped pores plays a decisive role because the N90 and N99 series also exhibit a strong difference in pore roundness but similar fatigue strength. The evaluation of the fracture surface analysis shows non-metallic aluminum-rich inclusions as the reason for cracking in all (except one) specimens built under nitrogen. These inclusions can also be found in argon specimens, but in addition, near-surface pores are responsible for around 50% of the crack initiation. This means that pores are not a problem in the specimens built under nitrogen but contribute greatly to the lower strengths of the specimens built under argon.
4. In all the studied conditions, a duplex microstructure with a predominant ferrite phase is obtained after complete processing. The phase compositions correlate very well with the determined nitrogen values of the different conditions: the two fully fused conditions Ar99 and N99 show a loss of nitrogen in the process and thus the lowest austenite content (43%), while the sample series N90 shows a nitrogen content slightly above the value of the powder material and thus shows the highest austenite content of 48%. The grain size distribution reveals an enormous grain refinement in the specimens with the shell core concept. The mean grain diameter of the ferrite grains has been reduced by half compared to the fully molten condition; in the case of the austenite grains, the reduction is not as pronounced.

In conclusion, the shell core concept is a promising process if certain rules are followed. For duplex steels, nitrogen is the process gas to favor in this approach, and the initial porosity should not exceed 10%. With regard to the samples built under argon, it would be useful in the next step to investigate a HIP-integrated heat treatment under pressure, as it can prevent the re-growth of argon porosity.

In a further step, the toughness and corrosion resistance of the different conditions should also be investigated, since these are crucial properties for the application of duplex steels.

## Figures and Tables

**Table 1:** Composition of the powder in comparison with the values according to DIN EN 10088-3.

	C	Si	Mn	P	S	Cr	Mo	Ni	N
AISI 318LN (in this study)	0.03	0.70	1.10	0.01	0.011	21.3	2.5	5.0	0.15
AISI 318LN (EN 10088-3)	0.03	<1.0	<2.0	<0.035	<0.015	21.0–23.0	2.5–3.5	4.5–6.5	0.10–0.22

**Table 2:** Process parameters for the PBF-LB/M process to achieve different relative densities.

Relative Density	Laser Power (W)	Hatch Distance (mm)	Layer Thickness (mm)	Scan Speed (m/s)
80%	175	0.60	0.05	1100
90%	175	0.25	0.05	1100
dense shell (>99%)	175	0.10	0.05	1100
>99%	200	0.10	0.05	1111

**Table 3:** Tabular representation showing the results of the rotating bending tests, evaluated with the SAFD©v5 software.

Name of Condition	N_K_	σ_D,50%_ (MPa)
Ar99	2,316,678	422
Ar90	1,203,421	301
Ar80	2,746,289	349
N99	839,720	454
N90	775,895	442
N80	1,717,642	305

**Table 5:** Mean grain diameter of ferrite and austenite.

	Ar80	Ar90	Ar99	N80	N90	N99
Grain diameter ferrite (µm)	15 ± 6	16 ± 8	32 ± 18	15 ± 6	14 ± 6	28 ± 15
Grain diameter austenite (µm)	10 ± 3	11 ± 4	13 ± 5	10 ± 4	10 ± 4	12 ± 5

**Table 6:** Results of pore analysis after HIP and heat treatment for the different conditions.

Name of Condition	Relative Density (%)	Circularity
Ar80	99.19	0.73
Ar90	99.34	0.60
Ar99 [40]	99.93 ± 0.03	0.96 (PBF-LB as-built condition)
N80	99.25	0.73
N90	99.43	0.71
N99 [40]	99.91 ± 0.04	0.91 (PBF-LB as-built condition)

**Table 7:** Results of nitrogen, argon, and oxygen measurements for the different conditions after HIP and heat treatment and the comparison to the powder used in this study.

Name of Condition	N (ppm)	Ar (ppm)	O (ppm)
Ar80	1419 ± 33	>20	502 ± 42
Ar90	1425 ± 28	10.63 ± 4.30	456 ± 21
Ar99 [40]	1287 ± 3	0.10 ± 0.02	-
N80	1407 ± 35	-	526 ± 37
N90	1554 ± 53	-	451 ± 27
N99 [40]	1345 ± 14	-	-
Powder Material	1500	<0.049	-

## Data Availability

The data presented in this study are available on request from the corresponding author.
